# Molecular mapping of QTLs for plant type and earliness traits in pigeonpea (*Cajanus cajan* L. Millsp.)

**DOI:** 10.1186/1471-2156-13-84

**Published:** 2012-10-08

**Authors:** Giriraj Kumawat, Ranjeet S Raje, Shefali Bhutani, Jitendra K Pal, Amitha SVCR Mithra, Kishor Gaikwad, Tilak R Sharma, Nagendra K Singh

**Affiliations:** 1 National Research Centre on Plant Biotechnology, Indian Agricultural Research Institute, New Delhi, 110012, India; 2 Division of Genetics, Indian Agricultural Research Institute, New Delhi, 110012, India; 3 Present address: Directorate of Soybean Research, Indore, Madhya Pradesh, 452001, India

**Keywords:** Earliness, Pigeonpea, Plant height, QTL mapping, SSR marker, SNP marker

## Abstract

**Background:**

Pigeonpea is an important grain legume of the semi-arid tropics and sub-tropical regions where it plays a crucial role in the food and nutritional security of the people. The average productivity of pigeonpea has remained very low and stagnant for over five decades due to lack of genomic information and intensive breeding efforts. Previous SSR-based linkage maps of pigeonpea used inter-specific crosses due to low inter-varietal polymorphism. Here our aim was to construct a high density intra-specific linkage map using genic-SNP markers for mapping of major quantitative trait loci (QTLs) for key agronomic traits, including plant height, number of primary and secondary branches, number of pods, days to flowering and days to maturity in pigeonpea.

**Results:**

A population of 186 F_2:3_ lines derived from an intra-specific cross between inbred lines ‘Pusa Dwarf’ and ‘HDM04-1’ was used to construct a dense molecular linkage map of 296 genic SNP and SSR markers covering a total adjusted map length of 1520.22 cM for the 11 chromosomes of the pigeonpea genome. This is the first dense intra-specific linkage map of pigeonpea with the highest genome length coverage. Phenotypic data from the F_2:3_ families were used to identify thirteen QTLs for the six agronomic traits. The proportion of phenotypic variance explained by the individual QTLs ranged from 3.18% to 51.4%. Ten of these QTLs were clustered in just two genomic regions, indicating pleiotropic effects or close genetic linkage. In addition to the main effects, significant epistatic interaction effects were detected between the QTLs for number of pods per plant.

**Conclusions:**

A large amount of information on transcript sequences, SSR markers and draft genome sequence is now available for pigeonpea. However, there is need to develop high density linkage maps and identify genes/QTLs for important agronomic traits for practical breeding applications. This is the first report on identification of QTLs for plant type and maturity traits in pigeonpea. The QTLs identified in this study provide a strong foundation for further validation and fine mapping for utilization in the pigeonpea improvement.

## Background

Pigeonpea (*Cajanus cajan* L. Millsp.) is an economically important grain legume of the tropical and subtropical regions of the world. It is grown extensively in India, South-East Asia, East Africa, Latin America and the Caribbean, where its plays important role in the food and nutritional security of the people. It is a major source of food protein to about 20% of the world population and is an abundant source of minerals and vitamins [1,2]. Further, it has significant usage as animal feed, fodder, firewood, thatching material and for improving soil structure and fertility. Globally, pigeonpea is cultivated in an area of 4.75 Mha with annual production of 3.68 MT and productivity of 706 kg/ha [3]. The average productivity of pigeonpea has remained very low and stagnant over the last five decades. A major constraint to pigeonpea productivity is the low genetic potential of pigeonpea varieties that have low harvest index, poor plant type, long crop duration and susceptibility to a host of biotic and abiotic stresses, besides socio-economic factors leading to poor crop management [4]. Exploitation of hybrid vigor, restructuring of plant type and early maturity are potential targets for increasing pigeonpea productivity per unit area and time [5,6]. Ideotype breeding is crucial for the suitability of a crop plant for modern farming practices, including traits for high harvest index and mechanical harvesting. It attempts to combine favorable QTLs for various component traits in a plant genotype [7]. Component traits of plant ideotype including plant height, number of branches, number of pods per plant and synchronous maturity play important role in shaping the plant architecture for high harvest index and mechanical harvesting. Early maturity is also needed for increasing cropping efficiency of the farming system. Although pigeonpea improvement through conventional breeding is going on, efforts for remodelling of pigeonpea plant type using genetic variability in the landraces and wild relatives with the help of modern biotechnological tools has not yet started.

The estimated size of the pigeonpea genome packed in its eleven chromosomes is about 853 Mb, which remained untouched by the genomics revolution for a long time [8,9]. Recently, a large number of simple sequence repeat (SSR) and single nucleotide polymorphism (SNP) markers have been developed under the Indo-US Agricultural Knowledge Initiative, creating opportunities for a large scale mapping of genes and quantitative trait loci (QTLs) for important agronomic traits [10-14]. Analysis of transcriptome and whole genome sequence using second generation high throughput sequencing technologies have made pigeonpea improvement amenable to molecular breeding [13,15-17]. Recently, an inter-specific linkage map of pigeonpea has been developed using 239 genomic SSR markers [14]. However, to our knowledge there is no published report on a dense intra-specific linkage map or QTL mapping of important agronomic traits in pigeonpea, except for limited studies using RAPD markers for tagging genes for *Fusarium* wilt resistance and plant type [18,19]. Earlier, SSR markers were used for the genetic diversity analysis of inbred lines and purity assessment of hybrids, but the level of polymorphism detected was very low [20]. The objective of present study was to develop an intra-specific high density framework linkage map of pigeonpea using genic SNP and SSR markers to identify QTLs for plant type and early maturity traits.

## Results

### Intra-specific molecular linkage map

**Figure 1:**
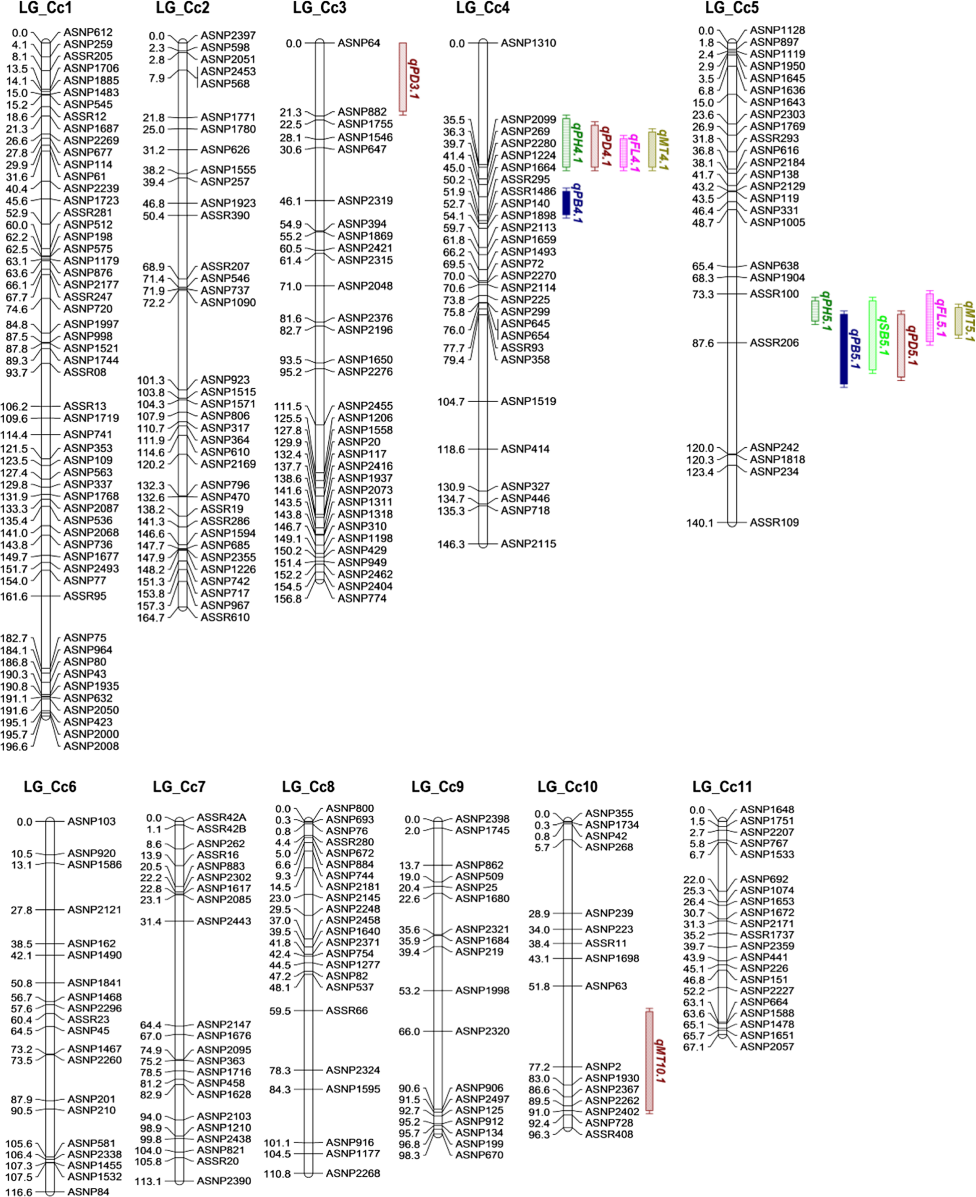
**Intra-specific linkage map of pigeonpea based on genic SNP and SSR markers showing location of QTLs for plant type and earliness traits (on the right side) identified in the Pusa Dwarf/ HDM4-1 mapping population.** Numbers on the left represent map positions in cM (Kosambi function). QTLs for each trait, with its abbreviation a part of the QTL name, is represented by different color/shading (PH, dark green; PB, blue; SB, light green, PD, dark red; FL, pink; MT, olive green).

Out of the total 595 SSR markers screened for parental polymorphism between Pusa Dwarf and HDM04-1, only 28 (4.7%) were found polymorphic that amplified 29 SSR loci (ASSR42 produced two loci). Details of these 28 SSR markers showing polymorphism in our mapping population are provided in Additional file 1. With the GoldenGate SNP assays, 160 SNPs (10.41%) were found polymorphic in the 1536-plex assay and 107 SNPs (13.93%) were found polymorphic in the 768-plex assay (details in Additional file 2 and Additional file 3, respectively). Overall 296 marker loci (10.17%) showed polymorphism and these were genotyped in all the 186 F_2_ plants. Total 280 marker loci (96.6%) showed good agreement with the expected 1:2:1 segregation ratio when analysed using *χ*^2^ test (P ≥0.01). Significant segregation distortion was observed for the remaining 16 (3.4%) marker loci. The direction of distortion for most of the loci was either towards the Pusa Dwarf allele or an excess of heterozygosity. A framework linkage map was constructed using all the 296 marker loci (Figure 1). Co-segregation analysis of markers resulted in the formation of eleven linkage groups at a cut-off recombination fraction of 0.35, which is equal to the haploid chromosome number for pigeonpea (*Cajanus cajan* L.). No marker was found unlinked after linkage group assignment and ordering. The linkage map of 296 marker loci covered a total observed genome length of 1406.7 cM, with an adjusted genome length of 1520.22 cM according to method 4 of Chakravarti et al. [21]. The average marker interval was 4.95 cM, providing a physical to genetic map ratio of 561.1 kb/cM taking a genome size of 853Mb [8]. The eleven linkage groups included 16 to 55 marker loci. The linkage groups were numbered LG_Cc1 to LG_Cc11 based on the descending order of their total map length in cM. The highest number of 55 loci was present on linkage group LG_Cc1 covering an adjusted map length of 203.8 cM, followed by LG_Cc2 consisting of 36 markers covering an adjusted map length of 174.1 cM and so on (Figure 1, Additional file 4). The shortest linkage group LG_Cc11 possessed 21 markers with an adjusted map length of 73.81 cM. The lowest number of 16 loci was mapped on the linkage group LG_Cc10 with an adjusted map length of 109.1 cM. There were only three gaps of larger than 30 cM in the genome, one each on LG_Cc4, LG_Cc5 and LG_Cc7, with map distance of 35.5, 32.5 and 33 cM, respectively. Majority of the loci with distorted segregation (9/16 loci) were clustered on the linkage group LG_Cc4. The remaining seven loci with distorted segregation were randomly distributed on five other linkage groups, suggesting that it may be due to a chance factor. The recommended marker density for genome wide QTL scanning is an average marker interval of less than 10 cM [22]. Thus the pigeonpea genetic map constructed in the present study with an average marker interval of 4.95 cM was quite suitable for the mapping of QTLs for plant type and earliness traits.

### Phenotypic variation for the plant type and earliness traits

**Figure 2:**
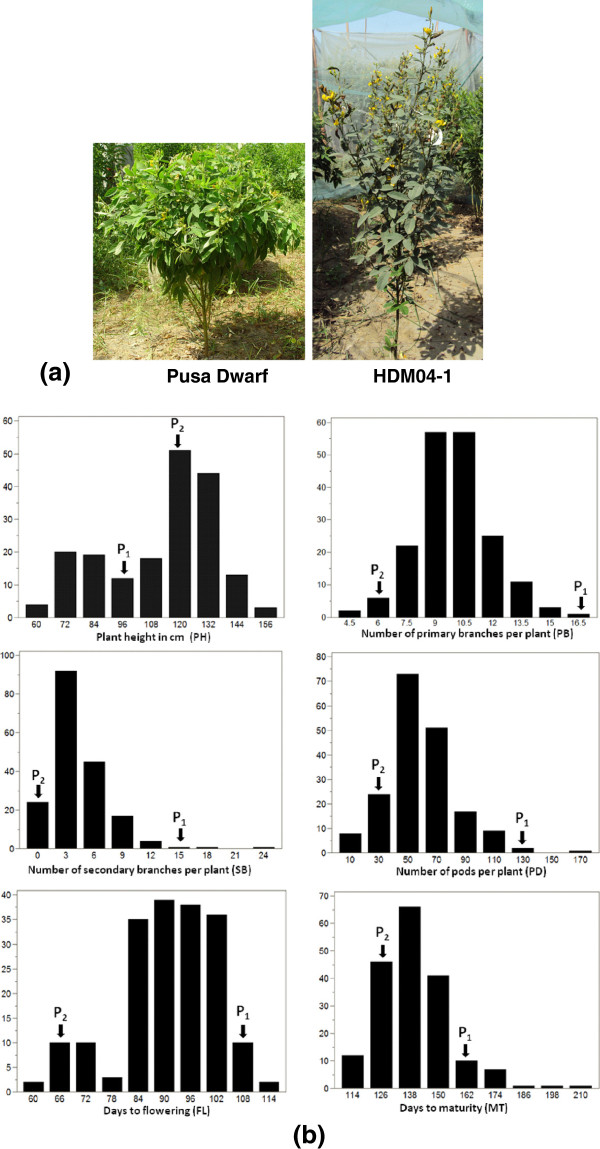
**(a) Field photographs of the parental genotypes Pusa Dwarf and HDM04-1; (b) Frequency distribution patterns of six plant architecture and earliness related traits in F**_**2:3 **_**population derived from cross between Pusa Dwarf and HDM04-1.** P_1_ = Pusa Dwarf, P_2_ = HDM04-1.

Phenotypic evaluation of the plant type and earliness traits showed significant variability in the Pusa Dwarf/ HDM04-1 mapping population (Table 1, Figure 2b). All the traits varied widely and their skewness values were less than 1.0, except for MT and SB. Frequency distribution for plant height and days to flowering showed bimodal distribution patterns, indicating involvement of major genes, whereas PB, PD and MT showed normal distribution patterns, suggesting involvement of multiple genes (Figure 2b). Transgressive segregation beyond both the parental means was observed for all the six traits, except for PB which did not show values higher than Pusa Dwarf. Pearson’s correlation coefficients (*r*) were calculated to see the relationships among various plant type and earliness traits. Significant positive correlations (*P* <0.01) were observed for all the pair wise combinations of traits, except for PH which was significantly correlated with PB and FL only (Table 2). Highly significant positive correlations were observed for all the pair wise combinations of PB, SB, PD, FL and MT, which may be due to close linkage of genes for these traits or pleiotropic effect of common gene(s) controlling these traits.

**Table 1:** **Descriptive statistics of six agronomic traits in the parents and F**_**2:3 **_**population derived from cross between Pusa Dwarf and HDM04-1**

**Trait**	**Parents**		**F**_**2:3 **_**Families**		
	**Pusa Dwarf**	**HDM04-1**	**Range**	**Mean ± SD**	**Skewness**
PH	87	119	59-160	112.12 ± 22.67	−0.552
PB	19	5	4-16.6	9.88 ± 1.95	0.199
SB	13	0	0-25	4.25 ± 3.24	2.33
PD	120	24	6.6-170.9	58.4 ± 23.9	0.853
FL	106	66	59-116	90.95 ± 11.17	−0.623
MT	158	116	110-208	140.09 ± 15.31	1.223

PH = plant height in cm; PB = number of primary branches/plant; SB = number of secondary branches/plant; PD = number of pods/plant; FL = days to flowering; MT = days to maturity.

**Table 2:** **Pearson’s correlation coefficients among the six plant type and earliness traits analyzed in the Pusa Dwarf /HDM04-1 F**_**2:3 **_**families**

Traits*	**PH**	**PB**	**SB**	**PD**	**FL**
**PB**	0.270**				
**SB**	−0.123	0.549**			
**PD**	−0.002	0.574**	0.535**		
**FL**	0.193**	0.517**	0.366**	0.631**	
**MT**	−0.112	0.409**	0.359**	0.545**	0.816**

*Trait abbreviations are shown in Table 1; **Significant at *P* < 0.01.

### QTL analysis

Thirteen significant QTLs were identified for the six agronomic traits analysed in this study using a full QTL model of the QTL Network software (Figure 1, Table 3). The proportion of phenotypic variation explained (PVE) by individual QTLs ranged from 3.18% to 51.4%. Ten of these QTLs showed major effects with PVE of more than 10% each. However, there could be overestimation of PVE due to relatively smaller size of the mapping population. There was clustering of QTLs in the same or adjacent marker intervals on the linkage groups LG_Cc4 and LG_Cc5. This was expected from the highly significant positive correlations among the traits and is likely due to pleiotropy or very tight linkage between the genes (Table 2). Significant epistatic interactions were observed only with the QTLs for number of pods per plant.

**Table 3:** **QTLs for plant type and earliness traits identified by QTL Network 2.1 using Pusa Dwarf / HDM04-1 F**_**2:3 **_**population**

**Trait**	**QTL Name**	**Marker interval**	**Position (cM)**	***F*value**	**PVE** **(%)**	**Additive effect**	**Dominance effect**
PH	*qPH4.1*	ASNP1310-ASNP2099	30.0	54.2	28.0	21.8	7.92
	*qPH5.1*	ASSR100-ASSR206	79.3	86.8	27.5	−20.14	14.94
PB	*qPB4.1*	ASNP1664-ASSR295	49.1	18.5	19.5	1.26	−0.03
	*qPB5.1*	ASSR206-ASNP242	87.6	10.9	11.1	0.81	0.19
SB	*qSB5.1*	ASSR100-ASSR206	85.3	11.0	10.4	1.64	−0.49
PD	*qPD3.1*	ASNP64-ASNP882	10.0	9.53	3.18	7.81	−13.20
	*qPD4.1*	ASNP2099-ASNP269	35.5	24.2	16.5	17.75	11.24
	*qPD5.1*	ASSR100-ASSR206	85.3	31.3	18.9	15.86	−7.35
FL	*qFL4.1*	ASNP1310-ASNP2099	34.0	84.0	51.4	14.4	5.3
	*qFL5.1*	ASSR100-ASSR206	79.3	26.3	8.7	5.54	−2.8
MT	*qMT4.1*	ASNP2099-ASNP269	35.5	38.0	22.6	11.72	3.10
	*qMT5.1*	ASSR100-ASSR206	81.3	49.9	25.9	13.09	−6.28
	*qMT10.1*	ASNP2262- ASNP2402	89.5	8.94	3.19	−4.13	−3.61

### QTLs for plant type traits

Two major additive effect QTLs, *qPH4.1* and *qPH5.1*, were identified for plant height (Figure 1, Table 3). The *qPH4.1* was located on the linkage group LG_Cc4 in the marker interval ASNP1310-ASNP2099 with a peak *F*-value of 54.2 and PVE of 28.0%, whereas, *qPH5.1* was located on linkage group LG_Cc5 in the marker interval ASSR100-ASSR206 with a peak *F*-value of 86.8 and PVE of 27.5%. The *qPH4.1* showed positive additive effect indicating that the allele for increasing plant height at this locus was contributed by Pusa Dwarf, whereas *qPH5.1* showed a negative additive effect indicating that the allele for increasing plant height at this locus was contributed by HDM04-1. Thus, HDM04-1 has a dwarfing allele at the *qPH4.1* locus whereas Pusa Dwarf has a dwarfing allele at the *qPH5.1* locus.

Two major QTLs were identified for the number of primary branches per plant, namely *qPB4.1* located on the LG_Cc4 in the marker interval ASNP1664-ASSR295 and *qPB5.1* located on LG_Cc5 in the marker interval ASSR206-ASNP242 (Figure 1, Table 3). The *qPB4.1* showed a peak *F*-value of 18.5 and PVE of 19.5%, whereas *qPB5.1* showed a peak *F*-value of 10.9 and a PVE of 11.1%. Both *qPB4.1* and *qPB5.1* showed positive additive effects indicating that the alleles for high number of primary branches were contributed by Pusa Dwarf at both the loci. A major additive effect QTL for number of secondary branches per plant, *qSB5.1* was mapped on LG_Cc5 in the marker interval ASSR100-ASSR206 with peak *F*-value of 11 and PVE of 10.4%. The positive allele at this locus for high number of SB was also contributed by Pusa Dwarf.

Two major additive effect QTLs and one minor QTL were detected for the number of pods per plant (Figure 1, Table 3). The first QTL, *qPD4.1* was located on LG_Cc4 in the marker interval ASNP2099-ASNP269 with a peak *F*-value of 24.2 and PVE of 16.5%. The second QTL, *qPD5.1 was* located on LG_Cc5 in the marker interval ASSR100- ASSR206 with a peak *F*-value of 31.3 and PVE of 18.9%. Positive alleles for high pod number per plant at both these loci were contributed by the genotype Pusa Dwarf. In addition, a minor additive effect QTL *qPD3.1* was mapped on LG_Cc3 in the marker interval ASNP64-ASNP882 with peak *F*-value of 9.53 and PVE of 3.18%. Positive allele at this locus was also contributed by the variety Pusa Dwarf.

### QTLs for earliness traits

Early maturity of crop plants is crucial for increasing the cropping efficiency of a farming system. One major and a minor QTL were detected for days to flowering, namely *qFL4.1* located on LG_Cc4 in the marker interval ASNP1310-ASNP2099 and *qFL5.1* located on LG_Cc5 in the marker interval ASSR100-ASSR206 (Figure 1, Table 3). The *qFL4.1* was a highly significant QTL with peak *F*-value of 84.0 and PVE of 51.4%, whereas *qFL5.1* was a relatively minor QTL with PVE of 8.7%. Both the QTLs showed positive additive effect, therefore alleles for late flowering at both the loci were contributed by Pusa Dwarf. Two major additive effect QTLs and one minor QTL were identified for days to maturity. The two major QTLs, *qMT4.1* and *qMT5.1*, were mapped on LG_Cc4 and LG_Cc5, respectively. The *qMT4.1* was located in the marker interval ASNP2099-ASNP269 with a peak *F*-value of 38.0 and PVE of 22.6%. The *qMT5.1* was located in the marker interval ASSR100-ASSR206 with a peak *F*-value of 49.9 and PVE of 25.9%. In addition, a minor QTL for days to maturity, *qMT10.1* was mapped on LG_Cc10 in the marker interval ASNP2262- ASNP2402, with peak *F*-value of 8.94 and PVE of 3.19%. While the first two QTLs showed positive additive effects, indicating alleles for late maturity in Pusa Dwarf, the reverse was true for locus *qMT10.1* with allele for late maturity coming from HDM04-1.

### Epistatic interaction and pleiotropic effects of QTLs

**Figure 3:**
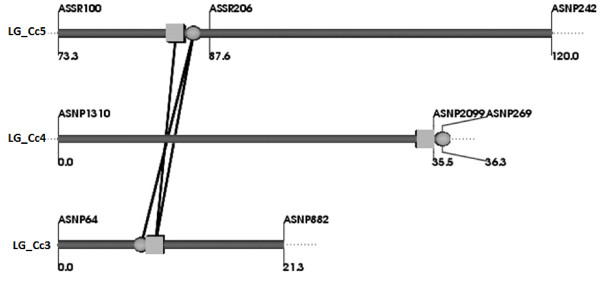
**Graphic representation of QTLs showing interaction effects for the number of pods per plant in Pusa Dwarf /HDM04-1 F**_**2:3 **_**population.** Circles represent QTLs with only additive effect while squares represents QTLs with only dominant effect. Connecting lines indicate significant interactions between the main effect QTLs.

Apart from the main effects, an epistatic interaction between different QTLs controlling the same trait was analyzed using a full-QTL model employing Markov Chain Monte Carlo algorithm of the QTL Network software. No significant epistatic effect was observed for PH, PB, SB, FL and MT. However, for number of pods per plant, QTL *qPD3.1* showed significant additive by additive, additive by dominance and dominance by dominance interactions with *qPD5.1* (Figure 3). The epistatic interactions between *qPD3.1* and *qPD5.1* contributed to 6.79% of the total phenotypic variance as indicated by the heritability estimate of the epistatic allele by QTL Network- 2.1 (Table 4). The thirteen QTLs identified in this study for the plant type and earliness traits were located in four genomic regions of pigeonpea chromosomes LG_Cc3, LG_Cc4, LG_Cc5 and LG_Cc10 (Figure 1). Ten of these QTLs were clustered in just two locations on the linkage groups LG_Cc4 and LG_Cc5. Since phenotypic values for most of the traits were significantly correlated with each other, the co-localization of QTLs was not entirely unexpected. To test whether this was due to pleiotropic effect of a single locus or close genetic linkage between different genes, the two QTL regions were analyzed for significance of pleiotropy using the Qgene software. The multiple trait multiple interval mapping (MMIM) function identified significant pleiotropic effects at both the QTL clusters as indicated by significantly high LOD of pleiotropy and LOD of the joint effect (Table 5).

**Table 4:** **Epistatic interactions between QTLs for number of pods per plant in the Pusa Dwarf/HDM04-1 F**_**2:3 **_**population of pigeonpea**

**Locus 1**	**Flanking markers**^**a**^	**LG (position)**^**b**^	**Locus 2**	**Flanking markers**^**a**^	**LG (position)**^**b**^	**Gene effect**^**c**^**(value)**	***P-*value**	**PVE**
*qPD3.1*	ASNP64-ASNP882	3 (10.0)	*qPD5.1*	ASSR100-ASSR206	5 (85.3)	AA (7.8)	0.015	1.58
						AD (-5.1)	0.251	3.74
						DD (17.54)	0.017	1.47

^a^ Markers flanking the of *F*-value peak interval for the QTL.

^b^ Location (in cM) of the highest point of *F*-value peak for the QTL.

^c^ AA, additive by additive; AD, additive by dominance; DD, dominance by dominance interaction.

**Table 5:** Pleiotropic effects of co-localized QTLs on two linkage groups identified by QGene 4.0 software

**Linkage** **group**	**Marker interval of QTL cluster (Map position)**	**Traits of co-located QTL**	**LOD of pleiotropy**	**LOD of joint effect**
LG_Cc4	ASNP1310-ASNP2099 (0-35 cM)	PH, PD, FL, MT	12	35
LG_Cc5	ASSR206-ASNP242 (87.6-120 cM)	PH, PB, SB, PD, FL, MT	39	480

## Discussion

Semi-dwarf plant type and synchronous early maturity are two very important agronomic traits for enhancing crop productivity per unit area and time by harnessing high harvest index and cropping efficiency. Grain legumes, particularly pigeonpea has lagged behind in the development of high yielding cultivars due to lack of basic information on the genomic regions and genes associated with these traits. In the present study, a dense molecular linkage map of pigeonpea was constructed using genic-SNP and genic-SSR markers to identify the QTLs or genomic regions associated with the plant type and early maturity traits. The genic-SNP markers showed significantly higher level of polymorphism (11.58%) than the genic-SSR markers (4.7%). Although this may be partly due to higher resolving power of the SNP assays, it will be prudent to use SNP markers for high density genetic mapping in crops like pigeonpea with narrow genetic base. This is due to high abundance of the SNP markers and high success rate of the GoldenGate SNP genotyping assays. In contrast, small allelic size differences of the SSR markers are not easily resolved by common agarose gel electrophoresis, although capillary electrophoresis can resolve these small size differences and produce higher polymorphism success rates [14]. The genic markers used in this study were developed from a comprehensive transcriptome assembly dataset; therefore this linkage map represents a random gene set of the pigeonpea genome [13]. Earlier a BES-SSR based integrated inter-specific genetic map of *C. cajan* × *C. scarabaeoides* has been reported that has 239 loci with a genome map length of 930.9 cM [14]. The inter–specific mapping population comprised of 79 F_2_ plants and a large proportion of the markers (63.5%) showed distorted segregation as compared to the present intra-specific population of 186 F_2_ plants with only 3.4% markers showing distorted segregation. The present map is the first high density intra-specific molecular linkage map of pigeonpea based on genic-SNP and genic-SSR markers and covers a much higher genome map length of 1520.22 cM. This is due to a larger population size and better pairing and crossing over between the chromosomes of two varieties of the same species. Furthermore, the present map represents expressed regions of the pigeonpea genome; therefore it will be highly useful for comparative genomics and synteny studies. However, genic markers represent mostly the euchromatic regions of the genome; hence heterochromatin and other repeat regions may be underrepresented, leading to large physical gaps between genic markers spanning these regions. There were three gaps of larger than 30 cM in the present genetic map, one each on LG_Cc4, LG_Cc5 and LG_Cc7, Presence of large gaps may lead to failure in detection of QTLs in the gap regions. Interestingly, large gaps were also observed in the recently developed genomic-SSR based consensus genetic map on LG8, LG10 and LG11 [14]. Although a direct comparison of the two maps was not possible due to lack of common markers, a non-uniform distribution of markers was apparent in both the maps.

This is the first report in pigeonpea on the mapping of QTLs for plant architecture and maturity time related traits which are very important for the development of superior varieties. Thirteen QTLs were identified, including ten QTLs having major effects with PVE of higher than 10%. The number of QTLs identified in a bi-parental mapping population depends on the number of trait controlling loci having contrasting alleles between the two parents [23]. The high PVE exhibited by the QTLs for many of the traits indicates involvement of segregating alleles of only a few critical genes leading to a large change in the plant architecture and maturity time of the two parents, particularly for PH and FL (Figure 2, Table 1). However, a possibility of the overestimation of QTL effects due to small population size, sometimes referred to as the Beavis effect, could not be ruled out in this preliminary investigation [24-26]. For further investigation of possible Beavis effects and validation of QTLs under different environments and genetic backgrounds, RIL mapping populations are being developed.

Plant height is an important agronomic and yield contributing trait for which two major QTLs were identified. Pusa Dwarf allele at the locus *qPH4.1* increased plant height by 21.8 cm, whereas HDM04-1 allele at the locus *qPH5.1* increased plant height by 20.1 cm. The two dwarfing genes will be very useful for the modification of plant type in pigeonpea. A high number of primary and secondary branches and number of pods per plant are also important yield contributing traits for which QTLs were collocated in the same marker interval. The QTLs *qPB4.1* and *qPB5.1* both showed positive additive effects with Pusa Dwarf alleles contributing to higher number of primary branches. The *qSB5.1* also showed positive additive effect with Pusa Dwarf allele contributing to a high number of secondary branches. The number of pods per plant directly contributes to higher grain yield and two major additive effect QTLs, *qPD4.1* and *qPD5.1* were identified, both showing positive additive effect with Pusa Dwarf alleles enhancing 17.75 pods and 15.86 pods per plant, respectively. A minor QTL on linkage group LG_Cc3, *qPD3.1* also showed positive additive effect with Pusa Dwarf allele increasing 7.81 pods per plant. Thus, Pusa Dwarf has several positive yield contributing traits that can be utilized in the pigeonpea improvement using marker-assisted breeding. It will be useful to combine the tallness allele of Pusa Dwarf at the *qPH4.1* locus with the dwarfing allele from Pusa Dwarf at the *qPH5.1* locus to obtain a semi-dwarf plant type with high number of primary branches, determinate growth habit and large number of pods per plant.

Pigeonpea has a large variation in the flowering and maturity time; therefore genetic mapping of these traits has direct implications for the development of short duration high yielding pigeonpea varieties. Two additive effect QTLs were identified for days to flowering (*qFL4.1* and *qFL5.1)* and alleles from the early flowering genotype HDM04-1 at these loci decreased the time of flowering by 14.4 days and 5.54 days, respectively. Three QTLs were identified for days to maturity, the two major loci *qMT4.1* and *qMT5.1* showed positive additive effects with alleles from Pusa Dwarf increasing the maturity time by 11.72 and 13.09 days, respectively. In addition, HDM04-1 allele at a minor locus *qMT10.1* increased the maturity time by 4.13 days. Hence, combining HDM04-1 alleles at the *qMT5.1* locus with Pusa Dwarf allele at the *qMT10.1* locus will reduce the days to maturity by about 17 days. Due to pleiotropic effects of the two QTL loci on height and maturity traits, it will be necessary to have the Pusa Dwarf allele at the *qMT.4.1* locus which is also associated with large number of primary branches and number of pods per plant. However, these are our preliminary observations, further validation of the QTLs at multiple environments and in different genetic background are needed for molecular breeding applications.

Epistatic interaction between *qPD3.1* and *qPD5.1* loci for number of pods was significant, accounting for 6.79% of the phenotypic variance. It illustrates the need for considering both additive and epistatic effects for devising effective molecular breeding strategy. Ten of the thirteen QTLs identified in this study were co-localized in just two genomic regions on linkage groups LG_Cc4 and LG_Cc5. The clustering of QTLs can be explained either by the presence of different tightly linked genes or by pleiotropic effects of a single regulatory gene [27]. Clustering of QTLs for different agronomic traits has been reported in many crops including soybean, common bean and rice [28-31]. Statistical analysis using Qgene software [32] devised for this purpose revealed that co-location of QTLs was due to significant pleiotropic effects at each of the two loci. The pleiotropy is possibly due to involvement of genes for the synthesis of phytohormones regulating common signaling pathways of the traits during plant development. Plant height was positively correlated with the number of primary branches, number of pods, flowering time and maturity time. As the flowering and maturity times increase, the plant gets more time for increased plant height and vegetative growth due to indeterminate growth habit.

Unfortunately, large genetic intervals were observed for some QTL regions, leading to poor resolution of closely linked QTLs and preventing accurate estimation of PVE. Although many of the QTLs identified in this study were mapped to small genomic regions of 5-15 cM (*qPH5.1, qPB4.1, qFL5.1, qMT5.1*) and were closely flanked by other polymorphic markers, their utilization in MAS is possible after validation in different genetic backgrounds and environments. Therefore, QTL validation and fine mapping is the next step towards successful application of these findings in MAS.

## Conclusions

The work presented here describes the first intra-specific dense molecular linkage map of pigeonpea largely based on genic-SNP markers. It is also the first report on QTL mapping of agronomically important traits in pigeonpea, including plant height, number of branches and pods per plant and maturity time. These results provide strong basis for further investigation on validation and fine mapping of the identified QTLs for plant type and earliness traits, which would help in developing high-yielding early-maturing varieties of pigeonpea for food and nutritional security in the semi-arid tropical and sub-tropical countries.

## Methods

### Plant material

A population of 186 F_2:3_ lines derived from a cross between inbred lines ‘Pusa Dwarf’ and ‘HDM04-1’ was used. Pusa Dwarf has a compact dwarf plant type, determinate growth habit, late maturity and higher number of primary and secondary branches and number of pods per plant as compared to HDM04-1 which has a medium plant height, indeterminate growth habit, early maturity with lower numbers of primary branches and pods per plant (Figure 2a, Table 1). Original seeds of the two parental lines were obtained from the Indian Agricultural Research Institute, New Delhi.

### Experimental details and trait phenotyping

The 186 F_2:3_ families and the two parental lines were planted in single rows with 60×20 cm spacing in two replications in a randomized block design (RBD) at the research farm of Indian Agricultural Research Institute, New Delhi in June 2009. The field management followed standard agricultural practices. Observations were recorded on plant height (PH in cm), number of primary branches per plant (PB), number of secondary branches per plant (SB), number of pods per plant (PD), days to flowering (FL) and days to maturity (MT). Ten plants from the middle of each row were used for trait scoring. Days to flowering was scored as number of days from the date of sowing on which the first flower opened, whereas days to maturity was scored as number of days from the date of sowing when 80% of the pods turned yellow. All the plant type traits viz. PH, PB, SB and PD were recorded at maturity.

### Genotyping and construction of linkage maps

Genomic DNA was isolated from 2 g leaf sample of each of the F_2_ plants using cetyl trimethyl ammonium bromide (CTAB) method with minor modifications [33]. Total 595 SSR markers consisting of 550 genic-SSR [13] and 45 genomic-SSR [4,34] were used for polymorphism survey between the parental lines Pusa Dwarf and HDM04-1. The SSR markers were PCR amplified and separated by electrophoresis in high resolution 4% Metaphor agarose gels or 8% polyacrylamide gels. Multiplexed genic-SNP markers, including a 1536-plex and a 768-plex assays employing Illumina’s GoldenGate technology, were used for SNP genotyping of the F_2_ population. The SNPs were identified by aligning high quality transcriptome shotgun assembly (TSA) sequence contigs of pigeonpea varieties Asha (35,204 contigs, NCBI Ac. no. EZ647865-EZ683068) and UPAS 120 (30,147 contigs, NCBI Ac. no. EZ617718-EZ674864) [13], using Lasergene SeqMan Pro^Tm^ Version 8.0.12 software and designated ASNP1-ASNP2498. From this SNP list, two multiplex assays of 1536-plex and 768-plex were designed using Illumina’s GoldenGate assay design tool ( http://www.illumina.com). Genomic DNAs (250 ng per sample) were used as templates for the SNP genotyping using Illumina's bead array technology according to the manufacturer's protocol [35]. Allelic segregation at each of the marker loci was analysed for deviation from the expected 1:2:1 ratio in the F_2_ population using *χ*^2^ test and linkage maps were constructed using Mapdisto version 1.7.5 software [36]. Seriation-II method was used for ordering of the loci with the criteria SARF for ordering and ripple. A cut-off recombination value of 0.35 and threshold LOD score of 3.0 was used. The Kosambi function was used for the estimation of map distances [37]. Adjusted genetic map lengths (in cM) were calculated using method 4 of Chakravarti et al. [20,38].

### Statistical analysis and QTL mapping

Statistical analysis of the phenotypic data was performed using SAS software (SAS Institute Inc. USA). Pearson’s correlation coefficients were calculated for pairwise trait combinations. The QTL analysis was performed using means of the phenotypic trait values from two replications with QTL Network version 2.1 software employing the full-QTL model [39]. The test window size was set to 5 cM with a walk speed of 2 cM and a cut-off probability of 0.05 for deciding the significance of the QTL. *F-*value threshold for declaring a QTL significant was determined by permutation tests using 1,000 reiterations [40]. The QTL main effects and their epistasic interactions were analysed by the full-QTL model using a Markov Chain Monte Carlo algorithm. Graphical presentation of the linkage groups (LGs) and the QTLs were obtained using MapChart version 2.2 [41]. Pleiotropy of co-localized QTLs was tested by multiple trait multiple interval mapping (MMIM) function of the Qgene 4.3.8 software [32].

## Abbreviations

BLAST: Basic local alignment search tool; cM: CentiMorgan; EST: Expressed sequence tag; EST-SSR: An SSR obtained from a cDNA sequence located in EST; ESTP: Expressed sequence tag polymorphism; GO: Gene Ontology; LG: Linkage group; LOD: Log10 of the likelihood odds ratio; NCBI: National Center for Biotechnology Information; PCR: Polymerase chain reaction; QTL: Quantitative trait locus; SNP: Single nucleotide polymorphism; SSR: Simple sequence repeat.

## Competing interests

The authors declare that they have no competing financial interests.

## Authors’ contributions

GK carried out SSR genotyping, linkage map construction, phenotyping, QTL mapping and drafted the manuscript. RSR, GK, KG and TRS contributed to the design of the study and mapping population development. SB developed SNP GoldenGate assays. JKP and ASVCRM carried out SNP genotyping. NKS conceived and supervised the study as well as finalized the manuscript. All authors have read and approved the final version of the manuscript.

## Supplementary Material

Additional file 1 Table showing details of 28 polymorphic SSR markers used in the construction of linkage map of pigeonpea from the Pusa Dwarf/HDM04-1 population. Click here for file

Additional file 2 Table showing SNP positions [in bracket] and flanking sequences (about 50 bases each side of the SNPs) in the 1536-plex Illumina Goldengate assay, with the SNP Id, TSA (transcript shotgun assembly) contig Id of the sequence from variety 'Asha' (NCBI GenBank Ac. no. EZ647865-EZ683068) and SNP quality scores based on Illumina SNP assay design tool. Click here for file

Additional file 3 Table showing SNP positions [in bracket] and flanking sequences (about 50 bases each side of the SNPs) in the 768-plex Illumina Goldengate assay, with the SNP Id, TSA (transcript shotgun assembly) contig Id of the sequence from variety 'Asha' (NCBI GenBank Ac. no. EZ647865-EZ683068) and SNP quality scores based on Illumina SNP assay design tool. Click here for file

Additional file 4 Table showing observed and adjusted map lengths of the 11 linkage groups of pigeonpea. Click here for file

## References

[B1] Saxena KB Kumar RV Rao PV Basra AS, Randhawa IS Pigeonpea nutrition and its improvement Quality Improvement in Field Crops 2002 New York: Food Products Press 227 260

[B2] Thu TT Mai TTX Dewaele E Farsi S Tadesse Y Angenon G Jacobs M *In vitro* regeneration and transformation of pigeonpea (*Cajanus cajan* (L.) Millsp.) Mol Breed 2003 11 159 168 10.1023/A:1022497811702

[B3] FAOSTAT 2012 http://faostat.fao.org

[B4] Odeny DA Jayashree B Ferguson M Hoinsington D Crouch J Gebhardt C Development, characterization and utilization of microsatellite markers in pigeonpea Plant Breed 2007 126 130 136 10.1111/j.1439-0523.2007.01324.x

[B5] Saxena KB Sharma D Nene YL, Hall SD, Sheila VK Pigeonpea genetics The pigeonpea 1990 Wallingford (UK): CAB International 137 158

[B6] Saxena KB Genetic improvement of pigeonpea—a review Trop Plant Biol 2008 1 159 178 10.1007/s12042-008-9014-1

[B7] Wu RL Genetic mapping of QTLs affecting tree growth and architecture in Populus: implication for ideotype breeding Theor Appl Genet 1998 96 447 457 10.1007/s001220050761 24710884

[B8] Greilhuber J Obermayer R Genome size variation in Cajanus cajan (Fabaceae): a reconsideration Plant Syst Evol 1998 212 135 141 10.1007/BF00985225

[B9] Yang S Pang W Harper J Carling J Wenzl P Huttner E Zong X Kilian A Low level of genetic diversity in cultivated pigeonpea compared to its wild relatives is revealed by diversity arrays technology (DArT) Theor Appl Genet 2006 113 585 595 10.1007/s00122-006-0317-z 16845522

[B10] Varshney RK Close TJ Singh NK Hoisington DA Cook DR Orphan legume crops enter the genomics era Curr Opin Plant Biol 2009 12 202 210 10.1016/j.pbi.2008.12.004 19157958

[B11] Varshney RK Penmetsa RV Dutta S Kulwal PL Saxena RK Datta S Sharma TR Rosen B Carrasquilla-Garcia N Farmer AD Dubey A Saxena KB Gao J Fakrudin B Singh MN Singh BP Wanjari KB Yuan M Srivastava RK Kilian A Upadhyaya HD Mallikarjuna N Town CD Bruening GE He G May GD McCombie R Jackson SA Singh NK Cook DR Pigeonpea genomics initiative (PGI): an international effort to improve crop productivity of pigeonpea (*Cajanus cajan* L.) Mol Breed 2010 26 393 408 10.1007/s11032-009-9327-2 20976284 PMC2948155

[B12] Raju NL Gananesh BN Lekha P Jayashree B Pande S Hiremath PJ Byregowda M Singh NK Varshney RK The first set of EST resource for gene discovery and marker development in pigeonpea (*Cajanus cajan* L.) BMC Plant Biol 2010 10 45 10.1186/1471-2229-10-45 20222972 PMC2923520

[B13] Dutta S Kumawat G Singh BP Gupta DK Singh S Dogra V Gaikwad K Sharma TR Raje RS Bandhopadhya TK Datta S Singh MN Bashasab F Kulwal P Wanjari K Varshney RK Cook DR Singh NK Development of genic-SSR markers by deep transcriptome sequencing in pigeonpea [*Cajanus cajan* (L.) Millspaugh] BMC Plant Biol 2011 11 17 10.1186/1471-2229-11-17 21251263 PMC3036606

[B14] Bohra A Dubey A Saxena RK Penmetsa RV Poornima KN Kumar N Farmer AD Srivani G Upadhyaya HD Gothalwal R Ramesh S Singh D Saxena K Kishor PB Singh NK Town CD May GD Cook DR Varshney RK Analysis of BAC-end sequences (BESs) and development of BES-SSR markers for genetic mapping and hybrid purity assessment in pigeonpea, (*Cajanus* spp.) BMC Plant Biol 2011 11 56 10.1186/1471-2229-11-56 21447154 PMC3079640

[B15] Dubey A Farmer A Schlueter J Cannon SB Abernathy B Tuteja R Woodward J Shah T Mulasmanovic B Kudapa H Raju NL Gothalwal R Pande S Xiao Y Town CD Singh NK May GD Jackson S Varshney RK Defining the transcriptome assembly and its use for genome dynamics and transcriptome profiling studies in pigeonpea (*Cajanus cajan* L.) DNA Res 2011 18 153 164 10.1093/dnares/dsr007 21565938 PMC3111231

[B16] Singh NK Gupta DK Jayaswal PK Mahato AK Dutta S Singh S Bhutani S Dogra V Singh BP Kumawat G Pal JK Pandit A Singh A Rawal H Kumar A Prashat GR Khare A Yadav R Raje RS Singh MN Datta S Fakrudin B Wanjari KB Kansal R Dash PK Jain PK Bhattacharya R Gaikwad K Mohapatra T Srinivasan R Sharma TR The first draft of the pigeonpea genome sequence J 2011 21 98 112 10.1007/s13562-011-0088-8 PMC3886394 24431589

[B17] Kudapa H Bharti AK Cannon SB Farmer AD Mulaosmanovic B Kramer R Bohra A Weeks NT Crow JA Tuteja R Shah T Dutta S Gupta DK Singh A Gaikwad K Sharma TR May GD Singh NK Varshney RK A comprehensive transcriptome assembly of pigeonpea (*Cajanus cajan* L.) using sanger and second-generation sequencing platforms Mol Plant 2012 1 9 10.1093/mp/ssr111 22241453 PMC3440007

[B18] Kotresh H Fakrudin B Punnuri SM Rajkumar BK Thudi M Paramesh H Lohithaswa H Kuruvinashetti MS Identification of two RAPD markers genetically linked to a recessive allele of a Fusarium wilt resistance gene in pigeonpea (*Cajanus cajan* L. Millsp.) Euphytica 2006 149 113 120 10.1007/s10681-005-9059-2

[B19] Dhanasekar P Dhumal KN Reddy KS Identification of RAPD markers linked to plant type gene in pigeonpea Indian J Biotechnol 2010 9 58 63

[B20] Chakravarti A Lasher L Reefer J A maximum likelihood method for estimating genome length using genetic linkage data Genetics 1991 128 175 182 2060775 10.1093/genetics/128.1.175 PMC1204446

[B21] Saxena RK Saxena K Varshney RK Application of SSR markers for molecular characterization of hybrid parents and purity assessment of ICPH 2438 hybrid of pigeonpea [*Cajanus cajan* (L.) Millspaugh] Mol Breed 2010 26 371 380 10.1007/s11032-010-9459-4

[B22] Doerge RW Mapping and analysis of quantitative trait loci in experimental populations Nat Rev 2002 3 43 52 10.1038/nrg703 11823790

[B23] Mackay I Powell W Methods for linkage disequilibrium mapping in crops Trends Plant Sci 2007 12 57 63 10.1016/j.tplants.2006.12.001 17224302

[B24] Beavis WD Paterson AH QTL Analyses: Power, precision and accuracy Molecular dissection of complex traits 1998 Boca Raton: CRC Press 145 162

[B25] Xu S Theoretical basis of the Beavis effect Genetics 2003 165 2259 2268 14704201 10.1093/genetics/165.4.2259 PMC1462909

[B26] Raghavan C Collard BCY Effect of small mapping population sizes on reliability of quantitative trait locus (QTL) mapping African J Biotechnol 2012 11 10661 10674

[B27] Aastveit AH Aastveit K Effects of genotype-environment interactions on genetic correlations Theor Appl Genet 1993 86 1007 1013 10.1007/BF00211054 24194010

[B28] Mansur LM Orf JH Chase K Jarvik T Cregan PB Lark KG Genetic mapping of agronomic traits using recombinant inbred lines of soybean Crop Sci 1996 36 1327 1336 10.2135/cropsci1996.0011183X003600050042x

[B29] Beattie AD Larsen J Michaels TE Pauls KP Mapping quantitative trait loci for a common bean (*Phaseolus vulgaris* L.) ideotype Genome 2003 46 411 422 10.1139/g03-015 12834057

[B30] Blair MW Iriarte G Beebe S QTL analysis of yield traits in an advanced backcross population derived from a cultivated Andean wild common bean (*Phaseolus vulgaris* L.) cross Theor Appl Genet 2006 112 1149 1163 10.1007/s00122-006-0217-2 16432734

[B31] Wang P Zhou G Cui K Li Z Yu S Clustered QTL for source leaf size and yield traits in rice (*Oryza sativa* L.) Mol Breed 2012 29 99 113 10.1007/s11032-010-9529-7

[B32] Joehanes R Nelson JC QGene 4.0, an extensible Java QTL-analysis platform Bioinforma 2008 24 2788 2789 10.1093/bioinformatics/btn523 18940826

[B33] Murray MG Thompson WF Rapid isolation of high molecular weight plant DNA Nucleic Acid Res 1980 8 4321 4325 10.1093/nar/8.19.4321 7433111 PMC324241

[B34] Odeny DA Jayashree B Gebhardt C Crouch J New microsatellite markers for pigeonpea (*Cajanus cajan* (L.) millsp.) BMC Res Notes 2009 2 35 10.1186/1756-0500-2-35 19284532 PMC2660351

[B35] Shen R Fan JB Campbell D Chang W Chen J Doucet D Yeakley J Bibikova M Wickham Garcia E McBride C Steemers F Garcia F Kermani BG Gunderson K Oliphant A High-throughput SNP genotyping on universal bead arrays Mutat Res 2005 573 70 82 10.1016/j.mrfmmm.2004.07.022 15829238

[B36] Lorieux M MapDisto: fast and efficient computation of genetic linkage maps Mol Breed 2012 30 1231 1235 10.1007/s11032-012-9706-y

[B37] Kosambi DD The estimation of map distance from recombination values Ann Eugen 1944 12 172 175

[B38] Echt CS Saha S Krutovsky KV Wimalanathan K Erpelding JE Liang C Nelson CD An annotated genetic map of loblolly pine based on microsatellite and cDNA markers BMC Genet 2011 12 17 21269494 10.1186/1471-2156-12-17 PMC3038140

[B39] Yang J Hu C Hu H Yu R Xia Z Zhu J QTLNetwork: mapping and visualizing genetic architecture of complex traits in experimental populations Bioinforma 2008 24 721 723 10.1093/bioinformatics/btm494 18202029

[B40] Doerge RW Churchill GA Permutation tests for multiple loci affecting a quantitative character Genetics 1996 142 285 294 8770605 10.1093/genetics/142.1.285 PMC1206957

[B41] Voorrips RE Mapchart: software for the graphical presentation of linkage maps and QTLs J Hered 2002 93 77 78 10.1093/jhered/93.1.77 12011185

