# Supplementary material for: Molecular mapping of QTLs for plant type and earliness traits in pigeonpea (Cajanus cajan L. Millsp.)
Source: BMC Genet. 2012 Oct 8;13:84. doi: 10.1186/1471-2156-13-84 (PMC3504571; doi:10.1186/1471-2156-13-84)
Supplement: Additional file 4 — Table showing observed and adjusted map lengths of the 11 linkage groups of pigeonpea. [file 1471-2156-13-84-S4.docx]

**Additional file 4:** Observed and adjusted map lengths of the eleven linkage groups of pigeonpea based on the Pusa Dwarf/HDM04-1 F_2_ mapping population

| **Linkage group** | **No. of mapped loci** | **Observed map length (cM)** | **Adjusted map length (cM)*** |
| --- | --- | --- | --- |
| LG_Cc1 | 55 | 196.6 | 203.88 |
| LG_Cc2 | 36 | 164.7 | 174.11 |
| LG_Cc3 | 32 | 156.8 | 166.91 |
| LG_Cc4 | 28 | 146.3 | 157.13 |
| LG_Cc5 | 25 | 140.1 | 151.77 |
| LG_Cc6 | 20 | 116.6 | 128.87 |
| LG_Cc7 | 22 | 113.1 | 123.87 |
| LG_Cc8 | 23 | 110.8 | 120.87 |
| LG_Cc9 | 18 | 98.3 | 109.86 |
| LG_Cc10 | 16 | 96.3 | 109.14 |
| LG_Cc11 | 21 | 67.1 | 73.81 |
| **Total** | **296** | **1406.7** | **1520.22** |

*Adjusted according to the method 4 of Chakravarti et al. (1991)
